# Quantification the cost of injuries

**DOI:** 10.1093/eurpub/ckac129.634

**Published:** 2022-10-25

**Authors:** J Haagsma

**Affiliations:** Department of Public Health, Erasmus MC, Rotterdam, Netherlands

## Abstract

The population health impact of injuries can be measured with a range of metrics. ranging from incidence and mortality to disability adjusted life years and cost-of-illness. Cost of illness gives insight into the societal burden of injury, and factors and characteristics that are associated with higher costs. Assessment of the economic burden of health care is important, because this information allows us to understand main cost drivers of health care and to monitor the impact of injury prevention and interventions. However, comparability of cost-of-illness is hampered by differences in included and excluded health care resources, the perspective and time horizon that is applied in the cost-of-illness studies and inclusion or exclusion of productivity costs. Productivity costs refer to that are caused by work absence. In our injury cost of injuries studies, we found that, among working age injury patients, productivity costs are a significant contributor to the total costs related to injury. We also found that there is a lot of variety in the health care and productivity costs in injury patients, and these costs are not solely dependent on injury severity. In this presentation, we will discuss data sources that can be used to assess the cost of illness of injuries, methodological choices that can be made and risk factors for high health care and high productivity costs.

